# Multivariable Logistic Regression Model: A Novel Mathematical Model that Predicts Visual Field Sensitivity from Macular Ganglion Cell Complex Thickness in Glaucoma

**DOI:** 10.1371/journal.pone.0104126

**Published:** 2014-08-18

**Authors:** Daisuke Shiba, Shin Hatou, Takeshi Ono, Shingo Hosoda, Sachiko Tanabe, Naoki Ozeki, Kenya Yuki, Masaru Shimoyama, Kazumi Fukagawa, Shigeto Shimmura, Kazuo Tsubota

**Affiliations:** 1 Department of Ophthalmology, Keio University School of Medicine, Tokyo, Japan; 2 Keisho-kai Iidabashi Eye Clinic, Tokyo, Japan; 3 Tanabe Eye Clinic, Kai City, Yamanashi, Japan; 4 Shimoyama Eye Clinic, Shizuoka City, Japan; Cardiff University, United Kingdom

## Abstract

**Purpose:**

To design a mathematical model that can predict the relationship between the ganglion cell complex (GCC) thickness and visual field sensitivity (VFS) in glaucoma patients.

**Design:**

Retrospective cross-sectional case series.

**Method:**

Within 3 months from VFS measurements by the Humphrey field analyzer 10-2 program, 83 eyes underwent macular GCC thickness measurements by spectral-domain optical coherence tomography (SD-OCT). Data were used to construct a multiple logistic model that depicted the relationship between the explanatory variables (GCC thickness, age, sex, and spherical equivalent of refractive errors) determined by a regression analysis and the mean VFS corresponding to the SD-OCT scanned area. Analyses were performed in half or 8 segmented local areas as well as in whole scanned areas. A simple logistic model that included GCC thickness as the single explanatory variable was also constructed. The ability of the logistic models to depict the real GCC thickness/VFS in SAP distribution was analyzed by the χ^2^ test of goodness-of-fit. The significance of the model effect was analyzed by analysis of variance (ANOVA).

**Results:**

Scatter plots between the GCC thickness and the mean VFS showed sigmoid curves. The χ^2^ test of goodness-of-fit revealed that the multiple logistic models showed a good fit for the real GCC thickness/VFS distribution in all areas except the nasal-inferior-outer area. ANOVA revealed that all of the multiple logistic models significantly predicted the VFS based on the explanatory variables. Although simple logistic models also exhibited significant VFS predictability based on the GCC thickness, the model effect was less than that observed for the multiple logistic models.

**Conclusions:**

The currently proposed logistic models are useful methods for depicting relationships between the explanatory variables, including the GCC thickness, and the mean VFS in glaucoma patients.

## Introduction

Glaucoma is a neurodegenerative disease associated with the progressive loss of retinal ganglion cells (RGCs). Visual field testing has shown that loss of RGCs leads to decreased sensitivity to light stimulation. The current structure-function relationship hypothesis states that there is a retinal ganglion cell (RGC) functional reserve or RGC redundancy in early clinical glaucoma. This hypothesis is consistent with the histological finding that as many as half of the RGCs and their axons may be lost before there is detectable visual function loss, as measured by standard automated perimetry (SAP) [1], [2]. Therefore, the loss of RGCs likely leads to the atrophy of the ganglion cell layer (GCL).

Newer versions of optical coherence tomography (OCT) that incorporate spectral-domain (SD) technology provide higher scan resolutions and scan speeds. It has been shown that this technique can measure the thickness of the three or four innermost layers in the macula that are atrophic in glaucoma [3]. A further OCT study additionally confirmed there was a reduced thickness in the innermost three to four layers in the retina of glaucoma patients [4]. Upon development of SD-OCT, one of the first areas measured was the thickness of the ganglion cell complex (GCC), which consists of the retinal nerve fiber layer, GCL, and the inner plexiform layer (IPL) [5].

The continued development and evolution of the structure measurement methods have assisted researchers in their attempts to link the structural measurements to the visual functions. Previous studies that examined the structure-function relationship in glaucoma have developed one-to-one models between the structure (e.g., GCC thickness) and function (visual field sensitivity) [6]–[11]. However, other parameters such as age or spherical equivalent may also have a significant relationship with the structure measurements, such as the NFL or GCC thicknesses. In addition, many of these previous studies were not able to report definitive findings due to limited patient backgrounds, and because these studies only evaluated a small number of parameters. Therefore, to expand and clarify our understanding of the structure-function relationship in glaucoma, multiple regression analysis of previously excluded parameters will need to be performed. The purpose of current study was to design a mathematical model that could use the GCC thickness and other parameters, such as age, sex, or spherical equivalent, to predict the SAP-determined visual field sensitivity (VFS).

The relationship between the SAP-determined VFS and the GCC thickness does not appear to be a simple linear pattern. VFS reaches a plateau and does not increase without limitation, even when the GCC is sufficiently thick. On the other hand, steep decreases in the VFS seem to occur after the GCC thickness falls below a certain threshold. In addition, regardless of the type of SAP used, there are minimum and maximum detectable limits of VFS. Because of these characteristics and restrictions, plots of the GCC thickness/VFS in SAP curves result in so-called sigmoid or z-shaped curves. Thus, these curves cannot be represented by a polynomial formula. Furthermore, with the exception of the GCC thickness, the effects of the other variants of VFS have yet to be definitively clarified. Therefore, the use of a nonlinear logistic analysis may be appropriate when examining such a complex pattern.

In this study, we analyzed the relationship between the local GCC thickness measured by SD-OCT and the VFS measured by SAP in eyes with glaucoma in conjunction with other explanatory variables determined by logistic regression analyses. We also evaluated the suitability of adapting this logistic model to the actual GCC thickness/VFS in SAP distribution.

## Patients and Methods

### Subjects

We retrospectively reviewed the medical records of patients with primary open-angle glaucoma who underwent both SAP by the Humphrey Visual Field Analyzer (Carl Zeiss Meditec, Dublin, CA) using the central 10-2 (HFA 10-2) program with a Swedish interactive threshold algorithm standard and macular GCC measurement by SD-OCT within a 3-month interval at Keisho-kai Iidabashi Eye Clinic, Tokyo, Japan. The HFA 10-2 inclusion period was from September 1, 2010 through August 31, 2011. All HFA 10-2 and macular GCC measurements by SD-OCT were performed during routine clinical practice. Glaucoma severity in the study population was determined by collecting the mean deviation (MD) values of HFA 24-2 within a year from the initial HFA 10-2 evaluation. Patients whose HFA 10-2 showed adequate reliability (fixation losses <33%, false positives and false negatives <20%) were included in the study. Analysis was performed only in the right eyes. All of the patients analyzed in the study underwent a complete ophthalmologic evaluation that included visual acuity testing, biomicroscopy of the anterior segment, intraocular pressure measurement by means of Goldmann applanation tonometry, and fundus examination. The inclusion criteria were a corrected visual acuity of 10/20 or better, a clear lens, and a normal retina. A 3-month testing window was selected to minimize the possibility of any glaucoma changes during the period between the structural and functional testing.

The diagnosis of glaucoma was based on the evaluations of three glaucoma experts (DS, NO, and KY). Although observation of optic disc rim thinning, including an enlarged vertical cup to disc ratio (>0.7), or asymmetry (>0.2) between two eyes was used for diagnosing glaucoma, the experts also used their clinical expertise with or without these two criteria to make the diagnosis. Exclusion criteria included a history of other ocular or neurologic diseases that could affect the structural or functional measurements, intraocular surgery, and use of systemic or topical medications that might affect the perimetric results.

Procedures followed the tenets of the Declaration of Helsinki, with the retrospective protocol approved by the Institutional Review Board of Keisho-kai Iidabashi Eye Clinic. Patient records and information were anonymized and de-identified prior to analysis.

### OCT measurements and GCC thickness analysis

Macular GCC analysis was measured by SD-OCT (RS-3000; Nidek, Inc., Gamagori, Japan), which acquires 53,000 A-scans per second. The GCC map was centered on the fovea, with the central macula covered by square grid of 30°×30°. The GCC map was created by acquiring 128 vertical B-scans that were composed of 512 A-scans. The 128 vertical B-scans were distributed in same interval within the 30° width. The GCC was automatically segmented from the internal limiting membrane to the outer IPL boundary.

**Figure 1:**
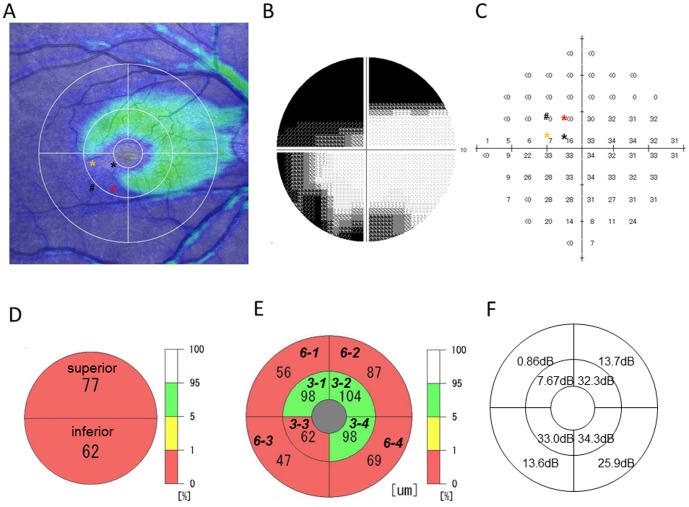
Methods of analysis for the macular ganglion cell complex thickness and standard automated perimetry. **A**) Representative ganglion cell complex (GCC) thickness map analyzed by RS-3000. Dividing lines and circles were drawn by the build-in software. **B**) Grayscale of the Humphrey visual field analyzer central 10-2 program in the same patient. **C**) Adjustment of the standard automated perimetry (SAP) test points to the GCC-map divided areas (GMDAs). Each inner GMDA (3-1 to 3-4) included three SAP test points (asterisks). Each asterisk of the SAP test points corresponds to the same color asterisk in the GCC map (top left). Each outer GMDA (6-1 to 6-4) includes 14 SAP test points. Note that the # is adjusted to the outer GMDA. **D**) Measurement of the GCC thickness in the superior and inferior areas. Numerical values in each area represent the mean GCC thickness (µm) of the area. **E**) Measurement of the GCC thickness in eight divided areas. These areas were named *3-1* to *6-4* GMDAs. Numerical values in each area represent the mean GCC thickness (µm) of the area. **F**) Mean visual field sensitivity is indicated in each GMDA.

The top panels of Figure 1 show representative data for the GCC thickness map analyzed by the RS-3000, the grayscale, and the SAP test point values determined by the HFA 10-2 program. The built-in software divided the GCC map into three concentric circles (central, middle, and lateral circles), and placed horizontal and vertical lines across the fovea (Figure 1A). The center of each circle was located at the fovea. The radii of the three concentric circles were 1.67°, 5°, and 10°, which respectively corresponded to 0.5, 1.5, and 3.0 mm in Gullstrand's schematic eye. The average thickness of the whole GCC scan field was measured as the mean GCC thickness of the area surrounded by the central and lateral circles. This division enabled the built-in software of the SD-OCT to measure the mean GCC thickness of the superior and inferior areas (Figure 1D) or the eight segmented areas (Figure 1E). The four areas surrounded by the central and middle circles were defined as the 3-1 to 3-4 GCC-map divided area (GMDA), while the four areas surrounded by the middle and lateral circles were defined as the 6-1 to 6-4 GMDA (Figure 1E). Images were excluded when the signal strength index was less than 7/10. When there were overt auto segmentation errors or obvious retinal abnormalities in the OCT, these images were also excluded.

### Adjustment of SAP test points to the GMDA

To ensure the analysis between the GCC thickness and the VFS is correct, data adjustment is required. Drasdo et al. [12] recently reported finding relatively large displacement in six human retinas. Based on their ganglion cell receptive field model in the human visual field, they were able to calculate the predicted lateral displacement of the ganglion cells. This adjustment method has been described in detail elsewhere [7], [11]. The predicted lateral displacement of the ganglion cells in the most centered visual field test points of the HFA 10-2 was 0.60 mm, while the displacement for the ganglion cells in the secondary centered visual field test points was 0.55 mm. The location of the most centered visual field test points and secondary centered visual field test points were 0.48 mm and 0.94 mm, respectively. Thus, the eccentricity in Gullstrand's schematic eye for the ganglion cells corresponding to the most centered visual field test points was 1.08 mm, while it was 1.49 mm for the ganglion cells corresponding to secondary centered visual field test points. Based on these estimates, the inner area surrounded by the central and middle circles of the GCC map includes the four most centered visual field test points and the eight secondary centered visual field test points. Similarly, the outer area surrounded by middle and lateral circles of the GCC map includes another 56 test points (Figure 1A, C). When the lateral displacement of the ganglion cells from the photoreceptor cells in each quadrant is taken into consideration, the inner three SAP test points (asterisks in Figure 1C) are included in the inner GMDA (i.e., 3-1, 3-2, 3-3, or 3-4). Similarly, adjustments of the outer 14 SAP test points, including the # point in Figure 1C, led to their placement in the outer GMDA (i.e., 6-1, 6-2, 6-3, or 6-4). The mean VFS in each GMDA was calculated as the average of the adjusted SAP test point values (Figure 1F).

### Multiple logistic model

To investigate the relationship between the mean VFS and the explanatory variables, which included GCC thickness (*x_1_*: µm), age (*x_2_*: years), sex (*x_3_*: male = *0*, female = *1*), and spherical equivalent (SE) of refractive errors (*x_4_*: diopters), we adapted a multiple logistic model to the mean VFS and explanatory variable data in order to create formula 1 as follows:
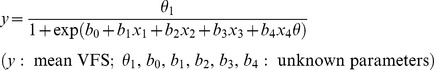
(1)


The above logistic regression analysis was solved using the Gauss-Newton method, with the estimators of the parameters θ_1_, and b_0_ to b_4_ obtained by JMP 8 software (SAS, Cary, NC). The *p*-values (*p*_0_ to *p*_4_) for each explanatory variable parameter were also obtained. Adaptation between the real VFS in SAP and the estimated VFS from the multiple logistic model was analyzed using the χ^2^ test of goodness-of-fit [13]. This approaches use the idea of comparing the observed number of individuals with each outcome to the number expected based on the logistic regression equation [13]. These observed (*O* = the real VFS in SAP) and expected (*E* = the estimated VFS from the logistic model) numbers are combined to form a goodness-of-fit chi square [13].
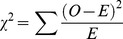



Large values of this test statistic indicate that the observed and expected results are very different, so that the regression equation has a poor fit to the data [13]. In contrast, small values of this test statistic indicate that the observed and expected results are similar, so the regression equation proves a good fit to the observations [13]. Alternatively, large *P* values (approaching 1) indicate a good fit and small *P* values (approaching 0) indicate a poor fit [13].

The distribution of the estimated VFS/GCC thickness dots from the model overlapped the real VFS/GCC thickness scatter plots. The significance of the model effect (i.e., predictability of the VFS from explanatory variables) was examined by an analysis of variance (ANOVA), followed by the calculation of the coefficient of determination (R^2^).

### Simple logistic model

To construct a simple logistic model that depicts the approximate relationship between the GCC thickness and the mean VFS value, some of the other explanatory variables in formula (1) were removed in order to create formula (2), which is as follows:
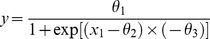
(2)


The θ_2_ parameter was defined as the GCC thickness at which the VFS decreased to half of the θ_1_, which represents the threshold of the obtained logistic curves. The θ_3_ parameter reflects the steepness of the slope of the curves. Using the GCC thickness and the mean VFS data, the regression formula and parameters (θ_1_, θ_2_, and θ_3_) were obtained by the Gauss-Newton method. The adaptation between the real VFS in SAP and the estimated VFS from the simple logistic model was analyzed by the χ^2^ test of goodness-of-fit. The significance of the model effect was examined by an ANOVA, followed by the calculation of the R^2^. Subsequently, the logistic curve of the estimated VFS obtained from the simple logistic model/GCC thickness was then drawn on the real VFS in the SAP/GCC thickness scatter plots.

### Statistical analysis

Scatter plotting, Gauss-Newton method, χ^2^ test of goodness-of-fit, ANOVA, and other statistical analyses were performed using Excel 2007 (Microsoft, Redmond, WA) and JMP 8 software. A *p* value of <0.05 was considered statistically significant.

## Results

### Patient characteristics

**Figure 2:**
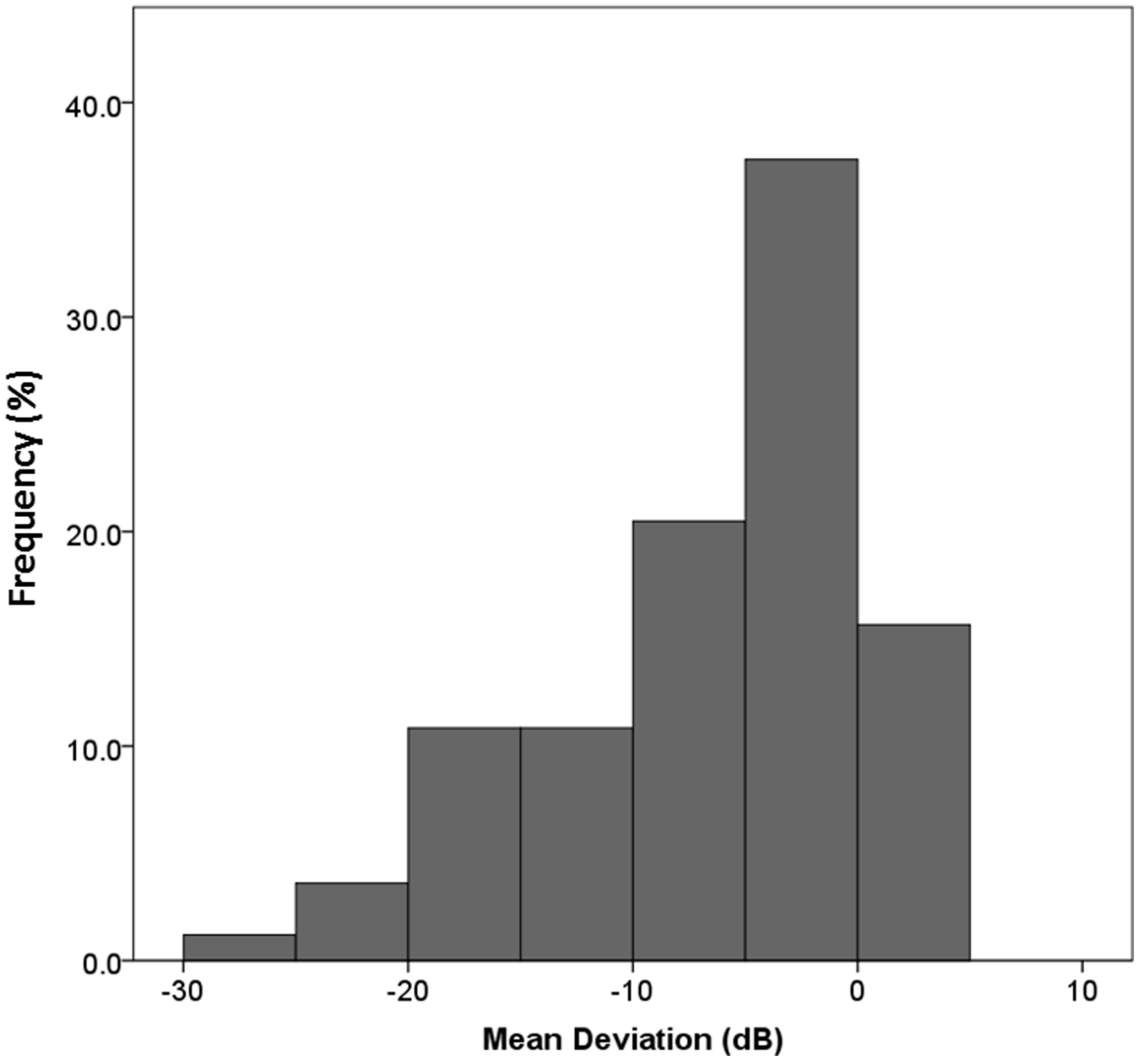
Histogram of glaucoma severity in the study population. The mean deviation values were measured by the central 24-2 program of the Humphrey visual filed analyzer. Patients with various stages of glaucoma were also included in the present study.

Out of the 95 candidate glaucoma eyes, 12 eyes were excluded due to overt segmentation error or other macular diseases such as idiopathic macular edema. As a result, the present study enrolled 83 glaucomatous eyes (48 men and 35 women). The raw data of enrolled eyes are presented in Table S1. The mean age of the enrolled patients was 55.9±11.9 years, with a mean SE of −3.8±3.4 diopters, a MD of the HFA 24-2 of −6.5±7.2 dB and a MD of the HFA 10-2 of −5.99±6.4 dB. In 16 of the enrolled eyes, a glaucomatous visual field defect was not observed in the HFA 24-2, even though the optic discs in these eyes were diagnosed as glaucomatous. Figure 2 shows the histogram of the MD values of the HFA 24-2. There was no statistically significant relationship between any of the data pairs for age, SE and MD and there was no statistically significant difference for the age, SE, and MD between the men and women (data not shown).

### Multiple logistic model parameters

Table 1 shows the θ_1_ and the b_0_ to b_4_ parameters, and the *p*-values for the explanatory variables obtained from the multiple logistic regression analysis. These values indicate the effect of the GCC thickness, age, sex, and SE of the refractive errors on the mean VFS in each area. The *p*-values revealed that in all of the areas analyzed, the GCC thickness exhibited the greatest effect, and was the most statistically significant parameter among all of the selected explanatory variables. While the other explanatory variables were statistically significant in several areas, these effects were less than that observed for the GCC thickness.

**Table 1:** The estimated parameters and *p*-values of the explanatory variables obtained from the multiple logistic regression analysis.

		θ_1_	b_0_ (intercept)	b_1_ (GCC thickness)	b_2_ (age)	b_3_ (sex)	b_4_ (SE)
whole field	estimators	32.354	5.308	−0.103	0.011	0.562	0.075
	*p*-value	**<0.001**	**<0.001**	**<0.001**	0.099	**0.003**	**0.017**
superior area	estimators	33.550	4.011	−0.088	0.019	0.163	0.075
	*p*-value	**<0.001**	**<0.001**	**<0.001**	**0.007**	0.444	**0.025**
inferior area	estimators	31.373	8.005	−0.150	0.012	0.631	0.065
	*p*-value	**<0.001**	**<0.001**	**<0.001**	0.195	**0.004**	0.115
3-1	estimators	34.354	2.099	−0.078	0.017	0.809	−0.047
	*p*-value	**<0.001**	**0.014**	**<0.001**	**0.038**	**0.002**	0.288
3-2	estimators	33.771	5.886	−0.083	−0.019	0.860	0.287
	*p*-value	**<0.001**	**<0.001**	**<0.001**	0.070	**0.004**	**<0.001**
3-3	estimators	33.617	6.322	−0.115	0.000	0.982	−0.023
	*p*-value	**<0.001**	**<0.001**	**<0.001**	0.976	**<0.001**	0.621
3-4	estimators	32.497	15.490	−0.254	−0.018	−0.023	−0.107
	*p*-value	**<0.001**	**<0.001**	**<0.001**	0.192	0.953	0.148
6-1	estimators	32.2688	7.330959	−0.15208	0.01438	0.193122	0.16887
	*p*-value	**<0.001**	**<0.001**	**<0.001**	0.156	0.461	**0.001**
6-2	estimators	31.84307	8.16703	−0.15263	0.030713	0.448431	0.24115
	*p*-value	**<0.001**	**<0.001**	**<0.001**	**0.003**	0.180	**<0.001**
6-3	estimators	31.78739	12.31516	−0.21936	−0.0143	0.840679	0.07381
	*p*-value	**<0.001**	**<0.001**	**<0.001**	0.305	**0.009**	0.237
6-4	estimators	30.48275	7.47111	−0.17642	0.046105	0.972715	0.08507
	*p*-value	**<0.001**	**<0.001**	**<0.001**	**<0.001**	**<0.001**	**0.031**

The estimators of b_2_, b_3_ and b_4_ in the whole scan area, superior area, inferior area, and most of the GMDAs were all positive quantities, with some of their *p*-values found to be statistically significant. This suggests that patients who are older, have hyperopia, or are female might have less VFS as compared to patients who are younger, have myopia, or are male. While several estimators of b_2_, b_3_ and b_4_ in GMDAs were inverted to negative quantities, none of their *p*-values were significant.

### Fitting the multiple logistic model on the scatter plots of the VFS/GCC thickness

**Figure 3:**
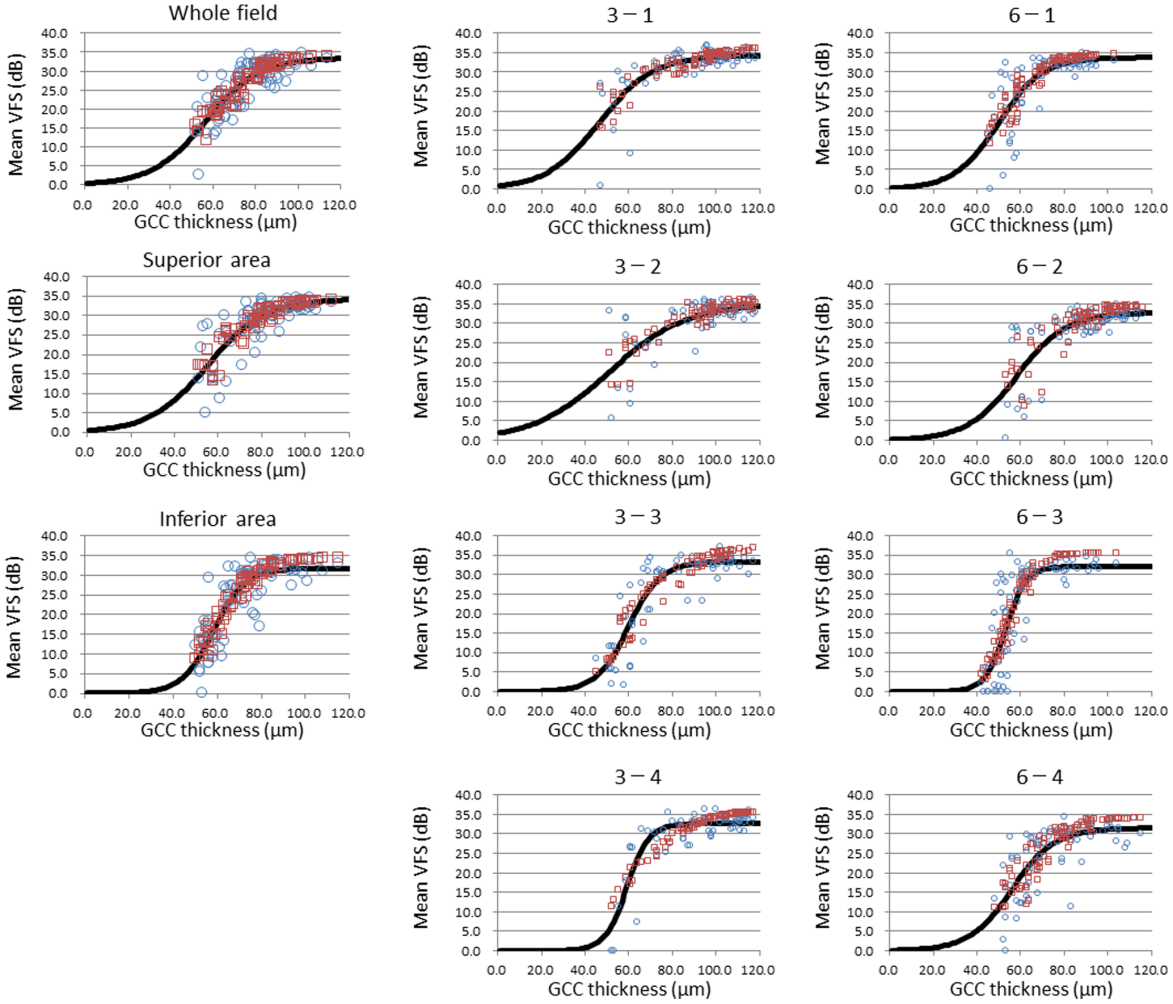
Visual filed sensitivity (VFS) versus ganglion cell complex (GCC) thickness. Scatter plots between the real VFS in standard automated perimetry and GCC thickness (blue circles) are shown. Distribution of the estimated VFS from the multiple logistic model/GCC thickness data overlapped on the same scatter plots (red squares). Curves of the simple logistic models (black curves) are also shown.

Figure 3 shows the scatter plots between the real VFS/GCC thickness (blue circles). The distribution of the estimated VFS/GCC thickness dots from the model (red squares) were found to overlap on the same scatter plots.

Table 2 shows the *p*-values from the χ^2^ test of goodness-of-fit, the *F*-and *p*-values of the ANOVA, and the R^2^ that were obtained for each of the adapted multiple logistic model solutions. The χ^2^ test of goodness-of-fit revealed that there was a good adaptation between the real VFS/GCC thickness and the estimated VFS/GCC thickness from the multiple logistic model in all scatter plots except in the 6-3 areas. The *F*-and *p*-values from the ANOVA revealed that the multiple logistic models of all areas were able to predict the VFS from the explanatory variables, with values statistically significant.

**Table 2:** The outcome of the χ^2^ test of goodness-of-fit, ANOVA, and the R^2^ of the multiple logistic models.

Area	χ^2^ test of goodness-of-fit	ANOVA		R^2^
	*p*-value	*F*-value	*p*-value	
whole field	0.9966	206.1	<0.0001	0.72
superior area	0.9979	189.8	<0.0001	0.70
inferior area	0.3876	267.3	<0.0001	0.77
3-1	0.9996	179.1	<0.0001	0.69
3-2	1.0000	180.5	<0.0001	0.69
3-3	0.1497	408.4	<0.0001	0.83
3-4	0.9995	252.6	<0.0001	0.76
6-1	0.2182	160.4	<0.0001	0.66
6-2	0.9928	290.7	<0.0001	0.78
6-3	**<0.0001**	179.4	<0.0001	0.69
6-4	0.7366	236.2	<0.0001	0.74

### Fitting the simple logistic model on the scatter plots of the VFS/GCC thickness

Table 3 shows the θ_1_, θ_2_, and θ_3_ parameters obtained from a simple logistic model, which depicts the approximate relationship between the GCC thickness and the VFS. Table 4 shows the *p*-values of the χ^2^ test of goodness-of-fit, the *F*- and *p*-values of the ANOVA, and the R^2^ that were obtained for the simple logistic model solution. Logistic curves of the estimated VFS/GCC thickness from the simple logistic model (black curves) overlap the scatter plots of the real VFS/GCC thickness (Figure 3). Although the obtained logistic curves visually appeared to run through the center of the real VFS/GCC thickness distribution on the scatter plots, the χ^2^ test of goodness-of-fit revealed that the estimated VFS from the simple logistic model failed to adapt the VFS in SAP in several areas (3-3, 6-1, 6-3, 6-4). These results suggest that other explanatory variables, such as age, sex, and SE, were necessary for adapting the estimated VFS to the real VFS in SAP. On the other hand, the *F*- and *p*-values from the ANOVA of the simple logistic model revealed that the simple logistic models in all areas were able to predict the VFS from the GCC thickness. Even though these predictions were found to be statistically significant, overall these effects were less than those observed for the multiple logistic models (Table 4).

**Table 3:** The estimated parameters of the explanatory variables obtained from the simple logistic regression analysis.

	θ_1_	θ_2_	θ_3_
whole field	33.479	56.629	0.080
superior area	34.303	54.999	0.076
inferior area	31.781	58.076	0.139
3-1	34.239	46.506	0.082
3-2	35.114	51.061	0.057
3-3	33.386	59.917	0.135
3-4	32.656	59.154	0.197
6-1	33.758	49.706	0.099
6-2	32.765	58.038	0.088
6-3	32.129	52.983	0.212
6-4	31.490	56.007	0.102

**Table 4:** The outcome of the χ^2^ test of goodness-of-fit, ANOVA, and the R^2^ of the simple logistic models.

Area	χ^2^ test of goodness-of-fit	ANOVA		R^2^
	*p*-value	*F*-value	*p*-value	
whole field	0.9606	144.8	<0.0001	0.64
superior area	0.9344	128.3	<0.0001	0.61
inferior area	0.0698	210.1	<0.0001	0.72
3-1	0.9990	115.7	<0.0001	0.59
3-2	0.9501	91.0	<0.0001	0.53
3-3	**0.0413**	295.7	<0.0001	0.78
3-4	0.9982	234.4	<0.0001	0.74
6-1	**0.0317**	110.1	<0.0001	0.58
6-2	0.0593	104.2	<0.0001	0.56
6-3	**<0.0001**	156.3	<0.0001	0.66
6-4	**0.0084**	106.2	<0.0001	0.57

## Discussion

Zeimer et al. [14] first reported that macular retinal thickness could be a criterion for the quantitative assessment of glaucoma. Axons, bodies, and dendrites of RGCs are believed to reside in the NFL, the GCL, and the IPL, respectively. Therefore, when detecting glaucomatous changes, it may be more accurate to measure the thickness of these specific layers rather than the total macular thickness. Ishikawa et al. [3] developed a macular segmentation algorithm and used time-domain OCT to demonstrate its ability to quantify the glaucomatous change in the inner macular thickness. To detect glaucomatous changes in their study, they analyzed four retinal segments, the NFL, GCC, GCL+IPL and the total retina, and demonstrated that the macular GCC thickness provided optimal glaucoma detection of the highest repeatability. Since RS-3000 SD-OCT can also measure the thickness of GCL+IPL, we attempted to create scatter plots using the GCL+IPL thickness that corresponded to the VFS. However, since the data distribution was scattered too widely, we could not perform a proper analysis (data not shown). Thus, this is the reason why we selected the GCC thickness in our present analysis. Based on the previous findings, we speculate that auto-segmentation between NFL and GCL would be more difficult to perform than that between the ILM and vitreous cavity, even when using SD-OCT, including RS-3000.

In the present study, the χ^2^ test of goodness-of-fit revealed that there was a good adaptation between the real VFS in SAP and the estimated VFS from the multiple logistic model in all of the scatter plots with the exception of the 6-3 area. In contrast, the estimated VFS from the simple logistic models were not able to adapt the VFS in SAP in a greater number of areas (3-3, 6-1, 6-3, 6-4). These results suggest that not only GCC thickness, but other explanatory variables, such as age, sex, and SE, are necessary for adapting the estimated VFS to the real VFS in SAP. Several studies have reported finding a structure and function relationship between the circumpapillary retinal nerve fiber layer thickness and the perimetric sensitivity in glaucoma patients [15]–[19]. A number of studies have also examined the macular thicknesses of GCC or GCL+IPL, and the perimetric sensitivity [6]–[11]. Kim et al. [6] reported finding a relationship between the macular GCC thickness, the MD, and the visual field index of the HFA 24-2 program. In their study, the relationships between the mean retinal nerve fiber layer/GCC thickness and the MD/visual field index were evaluated using regression analyses with a second-order polynomial formula. Furthermore, using a simple linear model based on previously published studies [7], [16]–[18], Sato et al. [11] demonstrated there was a relationship between the relative field sensitivity and the GCL+IPL thickness in glaucoma. In line with these previous reports, our current study also demonstrated that the effect of the GCC thickness on the VFS was greatest among all of selected explanatory variables. However, we also found that use of the single variable, GCC thickness, was insufficient and that other explanatory variables were necessary for properly adapting the estimated VFS to the real VFS.

Our current study used a nonlinear multiple logistic model in order to avoid the limitations associated with the first-, second-, or higher-order polynomial models. These types of models cannot accurately present the so-called sigmoid or z-shaped curves that result from the plateau of the VFS or detectable limits of SAP. On the other hand, the multiple logistic model can portray such shapes, and was able to adapt well to our observed data for the χ^2^ test of goodness-of-fit. However, since the multiple logistic model was rejected in the 6-3 area, there could be further unknown explanatory variables that may increase the error for fitting models in the 6-3 area. Therefore, additional studies designed to look for other explanatory variables will need to be undertaken in the future.

Previously there have been another report using models that GCC thickness reach a plateau when VFS were less than 20 dB [11]. This model stands on the assumption that there are layers that are not affected by glaucoma and the thickness of these layers are constant ( = GCC-thickness-plateau value) [20]. However, the GCC-thickness-plateau model has a severe defect that the curve of the model does not cover the GCC-thickness range less than GCC-thickness-plateau value. So in the case that GCC-thickness was less than GCC-thickness-plateau value, expected VFS value in this model would be mandatory regarded as 0 db, which might cause severe errors between expected and observed VFS values. Same problem would occur whenever certain lower limit of GCC-thickness values was placed in any models. So we avoid placing any limitation in GCC-thickness range so that expected VFS values would be obtained close to observed VFS values in any GCC-thickness values. The gradual slope of our simple and multiple logistic models in lower GCC-thickness range is the original merit, since it works to represent expected values of VFS at any GCC-thickness values. When compared presented simple logistic model and previous models, R^2^ values of presented simple logistic model exceeded that of previous models (Table 5), suggesting that presented logistic models functions have better predictability of the VFS from explanatory variables compared to linear model, second-order polynominal, third-order polynominal or GCC-thickness-plateau model.

**Table 5:** Comparison of R^2^ values between presented models and previous models.

	R^2^		
	whole field	superior area	inferior area
Simple logistic model (present study)	0.64	0.61	0.72
Multiple logistic model (present study)	0.72	0.70	0.77
Linear (Kim Na, et al [6])*	0.26	n/a	n/a
Second-order Polynominal (Kim Na, et al [6])*	0.30	n/a	n/a
Third-order Polynominal (Kim Na, et al [6])*	0.31	n/a	n/a
GCA-thickness-plateau model (Sato S, et al [11])†	0.38	0.24	0.45

GCA = ganglion cell layer and inner plexiform layer.

* R^2^ values between ganglion cell complex thickness and mean deviation value of 24-2.

† R^2^ values between GCA thickness and mean visual filed sensitivity by microperimetry.

In general, the logistic regression model is used for the dichotomous distribution. Concerning propriety of using logistic models for VFS/GCC thickness distributions, we consider VFS reflects the survival rate of functioning ganglion cells, which is probability of dichotomous variation, since VFS consists of sum of responses of the functioning ganglion cells. When total ganglion cells are healthy and functioning properly, VFS would be maximum value ( = θ_1_). As more and more ganglion cells suffer or die from glaucoma and lost their functions, survival rate of functioning ganglion cells decreases, and VFS/θ_1_ rate also decreases at the same time. When the assumption stands that VFS consists of sum of responses of the functioning ganglion cells, the survival rate of functioning ganglion cells and VFS/θ_1_ rate would be correspond, and VFS/θ_1_ would also be regarded as probability of dichotomous variation. We speculate this is why logistic models can be adapted to VFS/GCC thickness distributions so good. Unfortunately, it is just a speculation since there are no ways to measure the number of functioning ganglion cells in vivo. Further evaluations about relationship between VFS and survival rate of functioning ganglion cells are needed, and a method for measuring functioning ganglion cells is to be improved.

As the GCC thickness of the nasal area includes certain portions of the optic nerve fibers that originate from the ganglion cells in the temporal area, we were initially afraid that the fitness of the simple or multiple logistic models to the nasal area might be statistically rejected. However, our results showed these models were statistically accepted in the nasal areas. We speculate that these results are related to the anatomical characteristics of the retinal nerve fibers. Since the normal NFL thickness in the macula has been reported to be about one-half of the GCL+IPL thickness, the influence of the NFL thickness may not be great enough to increase the error of the fitness [4].

Since our multiple logistic model included a greater number of explanatory variables, its predictability was more accurate than that of the simple logistic model. However, the simple logistic model had some merits of its own, as it can visualize the logistic sigmoid curve that depicts the rough relationship between the GCC thickness and the mean VFS value. In addition, the thresholds of the obtained logistic curves were simply represented as the parameter θ_2_, which may be much easier to image than the multiple logistic model.

The analysis by these models demonstrated several different findings. First, multiple regression analysis revealed that patients who are older, have hyperopia or are female might have less VFS. Most previous studies that have examined the structure and visual field sensitivity relationship in glaucoma did not include such explanatory variables. Similarly, as our χ^2^ test of goodness-of-fit was rejected in the 6-3 area, our current study also may not have fully included all of the explanatory variables. Therefore, the mechanisms responsible for the effect of these explanatory variables on the VFS remain to be elucidated.

Since the plateau of the logistic curves suggests that the GCC thickness change in glaucoma occurs much earlier than the SAP sensitivity loss, SD-OCT measurements of the GCC thickness can be used to detect early glaucomatous damage. Thus, GCC thickness may be one of the most advantageous measurements for preperimetric or early perimetric glaucoma, which can be difficult to evaluate when using SAP.

In conclusion, after assessing explanatory variables such as the GCC thickness and examining the SAP sensitivity in patients with glaucoma, we were able to create a logistic model that can be used to depict the relationship between these variables. With the exception of the nasal-inferior-outer area (6-3), all of the multiple logistic models exhibited a good ability to fit the real GCC thickness/VFS in the SAP distribution to the χ^2^ test of goodness-of-fit. Our ANOVA results also showed that the models exhibited a statistically significant ability to predict the VFS from explanatory variables, which suggests that the model is a reliable method. Among all of the selected explanatory variables, the GCC thickness exhibited the greatest and statistically significant effect. ANOVA also revealed the simple logistic models could significantly predict the VFS. However, the adaptation and the effects of the multiple logistic models were found to be much better than those of the simple logistic models. Moreover, use of our model may be advantageous, especially when evaluating preperimetric or early perimetric glaucoma, which can be difficult to evaluate by SAP.

## Supporting Information

Table S1 **The raw data of enrolled eyes.**
(XLSX)
